# Empirically Integrating the Evidence for Different Predictive Coding Components Using Auditory False Perception

**DOI:** 10.1002/hbm.70211

**Published:** 2025-05-20

**Authors:** Feifan Chen, Anusha Yasoda‐Mohan, Colum Ó Sé, Sven Vanneste

**Affiliations:** ^1^ Lab for Clinical and Integrative Neuroscience, Trinity College Institute for Neuroscience, School of Psychology Trinity College Dublin Dublin Ireland; ^2^ Global Brain Health Institute Trinity College Institute for Neuroscience, Trinity College Dublin Dublin Ireland

**Keywords:** associative learning, false percept, internal model, prediction errors, strong priors, subjective confidence

## Abstract

Perception is a probabilistic estimation of the sensory information we receive at any given time and is shaped by an internal model generated by the brain by assimilating information over the life course. This predictive system in the brain has several components–(i) the internal model, (ii) the model‐based prediction called priors, (iii) the weighted difference between the prior and sensory input called prediction error (PE) and (iv) the weighted sum of the prior and input called perceptual inference. Until now, different studies have explored the independent components of this predictive coding system, and we, for the first time to our knowledge, integrate them. To do this, we induce a conditioned hallucination (CH) illusion by means of a multisensory integration paradigm and use this as a model to study the behavioral and electrophysiological responses to this experience. Additionally, we also probe their predictive coding system using a well‐established local–global auditory oddball paradigm. By comparing the behavioral and electrophysiological components of people more and less likely to perceive an illusion in the two paradigms, we observed that high perceivers place more confidence in their internal model and low perceivers in the sensory information. Furthermore, high perceivers were more sensitive than low perceivers to PEs that were generated by a change in the context of the sensory information, which served as a measure of a change in the internal model itself. As an exploratory analysis, we also observed that the objective likelihood of perceiving an illusion was corrected to the self‐reported likelihood of perceiving an illusion in a day‐to‐day setting, which disappears when controlled for the perceptual threshold. These results taken together start to give us an idea as to how a person's innate bias—either towards a learned model or external information may—affect their perception in a sensory context.

## Graphical abstract

This study integratively measured the independent components of the predictive coding system including priors, confidence, prediction errors (PEs) and perceptual inference. The results showed that subjects with higher confidence in priors are more likely to shape perception towards prior belief and are more sensitive to context‐driven PEs.

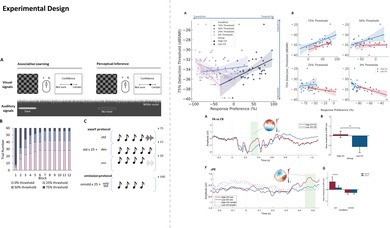

## 1 Introduction

Human perception is proposed to be a probabilistic inference combining prior beliefs of the environment and novel sensory information received at any given time (Friston 2010). This is theoretically explained by the Bayesian predictive coding framework wherein perception involves actively inferring the cause of sensations based on an internal model (Knill and Pouget 2004). The difference between the model‐based prediction (prior) and the sensory input, called prediction error (PE), updates the internal model to adapt to the changing environment (Friston 2010; Knill and Pouget 2004). This process happens at multiple hierarchical levels where the prior of the higher level aids in the selection of the input and the PE from the lower level serves as the input for the higher level (Arnal and Giraud 2012). The goal of perception is therefore to minimize the PE in this hierarchy.

From a probabilistic standpoint, perception is a weighted combination of the prior and the input, and the PE is a weighted difference between the two. If the input is more precise than the prior, the PE updates the prior. However, if priors are more precise than the input, the relatively “stronger” prior may bias perception, creating a false percept without an external source (Yon and Frith 2021; Corlett et al. 2019). Previous studies have shown that strong internal models that are backed up with confidence can facilitate false perceptions of the world (Corlett et al. 2019; Powers et al. 2017; Cassidy et al. 2018). Confidence is also suggested to reflect the internal feedback process that evaluates current sensory information in relation to prior knowledge about a multisensory world (Ptasczynski et al. 2022). From a predictive coding standpoint, confidence in a perceptual inference is proposed to (i) weight learning, (ii) integrate sensory information at early stages of processing, and (iii) be inversely related to sensory cortical responses, since the brain is proposed to rely less on the incoming input (Mulders et al. 2023).

Predictive coding, although a theoretical concept, is now gaining empirical traction. Different studies have examined the individual components of predictive coding that is priors, perceptual inference, PE, the role of confidence, etc. at a behavioral and neural level (Powers et al. 2017; Cassidy et al. 2018; Mohan et al. 2022; Sedley et al. 2019; Kafadar et al. 2022; Boldt et al. 2019). However, there is limited evidence as to how all these components are related to one another. Understanding how these different components interact with one another at a behavioral and electrophysiological level forms the basis of developing markers of targets for pharmacological and non‐pharmacological interventions, particularly given the accumulating evidence for different disorders to be aberrations of this foundational predictive system of the brain (Powers et al. 2017; Mohan et al. 2022; Yasoda‐Mohan, Chen, et al. 2024). In the current study, we use auditory illusion induced through the multisensory integration of visual and auditory input and the well‐established local‐global auditory oddball paradigm to study these different components of predictive coding in a within‐subject design to see how these components may interact with one another to produce a perceptual experience.

Priors are central to building a perceptual experience. Internal models can be built either by accumulating sensory evidence (Kelly et al. 2021) or through the implicit association between two stimuli (Powers et al. 2017). In the former, the accumulation of evidence is marked by a central positive potential (Kelly et al. 2021). The perceptual inference itself is proposed to be recalled from frontal regions (Cao et al. 2019) wherein the inferior frontal cortex resolves perceptual conflicts (Gao et al. 2023; Sugihara et al. 2006). When encountering uncertain sensory information, the brain relies on the internal model to make perceptual decisions leading to the generation of false perceptions. This is seen with both accumulation (Mc Keown et al. 2023) and association‐based paradigms (Powers et al. 2017). Particularly, Powers and Corlett showed that people making stronger prior associations between stimuli are more likely to perceive a false perception with high confidence (Powers et al. 2017). Our most recent study following this paradigm showed that these false inferences were associated with frontal activity, with patients with existing false perceptions making stronger internal models than those without (Yasoda‐Mohan, Chen, et al. 2024).

Parallelly, there is a wealth of research performed on assessing the neural correlates of error detection. The auditory oddball paradigm is a standardized way of investigating sensory errors in the auditory system (Squires et al. 1975). Subtracting the neural correlate of a standard predictable sequence from that of an unpredictable deviant generates a frontal negativity called mismatch negativity (MMN) around 100–250 ms, which forms the most basic neural correlate of PEs (Kirihara et al. 2020) and a later positive potential at 300 ms and later (P300) (Polich 2007). A more recent paradigm investigates these PEs from a hierarchical standpoint (Uhrig et al. 2014; Wacongne et al. 2011). Studies from our lab have shown that this paradigm produces two types of PE—a stimulus‐driven and context‐driven PE (sPE, cPE) which are generated by a difference in stimulus characteristics between the standard and deviant or when the same stimulus is presented with different probabilities (Yasoda‐Mohan, Faubert, et al. 2024). These PEs built in them mismatch and P300‐like potentials, showing complex auditory components at different levels of hierarchy.

In the current study, we will examine the interaction between different predictive coding components in forming a perceptual inference (i.e., inducing an auditory illusion) by investigating (i) how subjective confidence shapes priors, (ii) how priors build internal models, and (iii) how PEs manipulate internal models. The hypotheses for the study are (i) perception of illusions is driven by strong internal models that are built as a result of placing high confidence on the internal model during the model building phase, as proposed by Powers and Corlett, and (ii) compared to those who are less likely to perceive an illusion, people who are more likely to perceive an illusion have larger PEs that are driven by the context in which a stimulus is presented rather than the characteristics of the stimulus, also alluding to the formation of strong priors.

In the current study, generation of an internal model is facilitated through Powers and Corlett's implicit associative learning model (Powers et al. 2017). When the auditory environment becomes uncertain, this paradigm produces Conditioned Hallucinations (CH) in the auditory domain when cued by the visual stimulus. Examining the behavioral and neural responses during the learning phase and inference phase provides empirical estimates of priors, perceptual inference (i.e., illusion) and the role of confidence in shaping priors. To examine the role of PEs in forming strong priors and biasing perceptual inference, we apply the local–global auditory oddball paradigm (Yasoda‐Mohan, Faubert, et al. 2024). Combining the two paradigms together, we present a comprehensive empirical estimation of the different components of the predictive coding system. The results of this study can be used by future studies to expand on (i) other factors (e.g., personality traits, genetic and environmental risks) that can comprehensively describe the profile of those at risk of generating false perceptions in the general population, which can also serve as a preventative diagnosis for psychosis, and (ii) the complexity of the current paradigm to probe decision making at the perceptual and other higher‐order cognitive levels.

## 2 Methods

### 2.1 Ethics Statement

The study was approved by the School of Psychology Research Ethics Committee at Trinity College Dublin. All participants gave their written, informed consent per the EU General Data Protection Regulation 2016 and the Declaration of Helsinki.

### 2.2 Participants

The current study was powered based on the original study by Powers and Corlett, who recruited 30 controls and 30 patients (Powers et al. 2017). As we were interested only in controls, we recruited 44 participants. Exclusion criteria consisted of continuous phantom perception (tinnitus, verbal hallucination, phantom pain), chronic ear disorders (e.g., Menieres disease, ear infections, otosclerosis) and several neurological disorders such as tumors, mental disorders, and chronic headaches. All participants underwent a pure tone test where bilateral hearing thresholds at 500, 1000, 2000, 3000, 4000, 6000, and 8000 Hz were obtained. All participants were screened for hearing thresholds ≤ 30 dB HL and completed a set of questionnaires including Beck's Depression Inventory (BDI), Beck's Anxiety Inventory (BAI) which were applied to screen for mood disorders. Participants who scored > 11 on BAI, > 14 on BDI, and/or scored 2 or more on the suicide question in the BDI were excluded. Out of the 44 participants, two participants were unable to participate due to mild to moderate hearing loss (*n* = 1) or mild depression as reflected by a BDI score (*n* = 1). From the remaining 42 participants, three participants failed to correctly perform the experiments (sleeping, repeat the same response throughout the task) and two did not complete both paradigms. This left us with 37 participants (11 males and 26 females, Age = 21 ± 3.3 years) who were eligible for the study after screening for inclusion and exclusion criteria with the following demographics: audiogram values at 500 (Left: 17.30 ± 4.65 dB HL, Right: 14.73 ± 4.56 dB HL), 1000 (Left: 17.30 ± 4.65 dB HL, Right: 14.73 ± 4.56 dB HL), 2000 (Left: 16.89 ± 5.93 dB HL, Right: 14.73 ± 6.97 dB HL), 4000 (Left: 17.30 ± 6.41 dB HL, Right: 20.41 ± 4.91 dB HL), 6000 (Left: 17.97 ± 4.78 dB HL, Right: 22.57 ± 5.61 dB HL), and 8000 (Left: 17.97 ± 4.78 dB HL, Right: 22.57 ± 5.61 dB HL) Hz, BDI (4.24 ± 3.72), BAI (4.51 ± 4.86).

**FIGURE 1:**
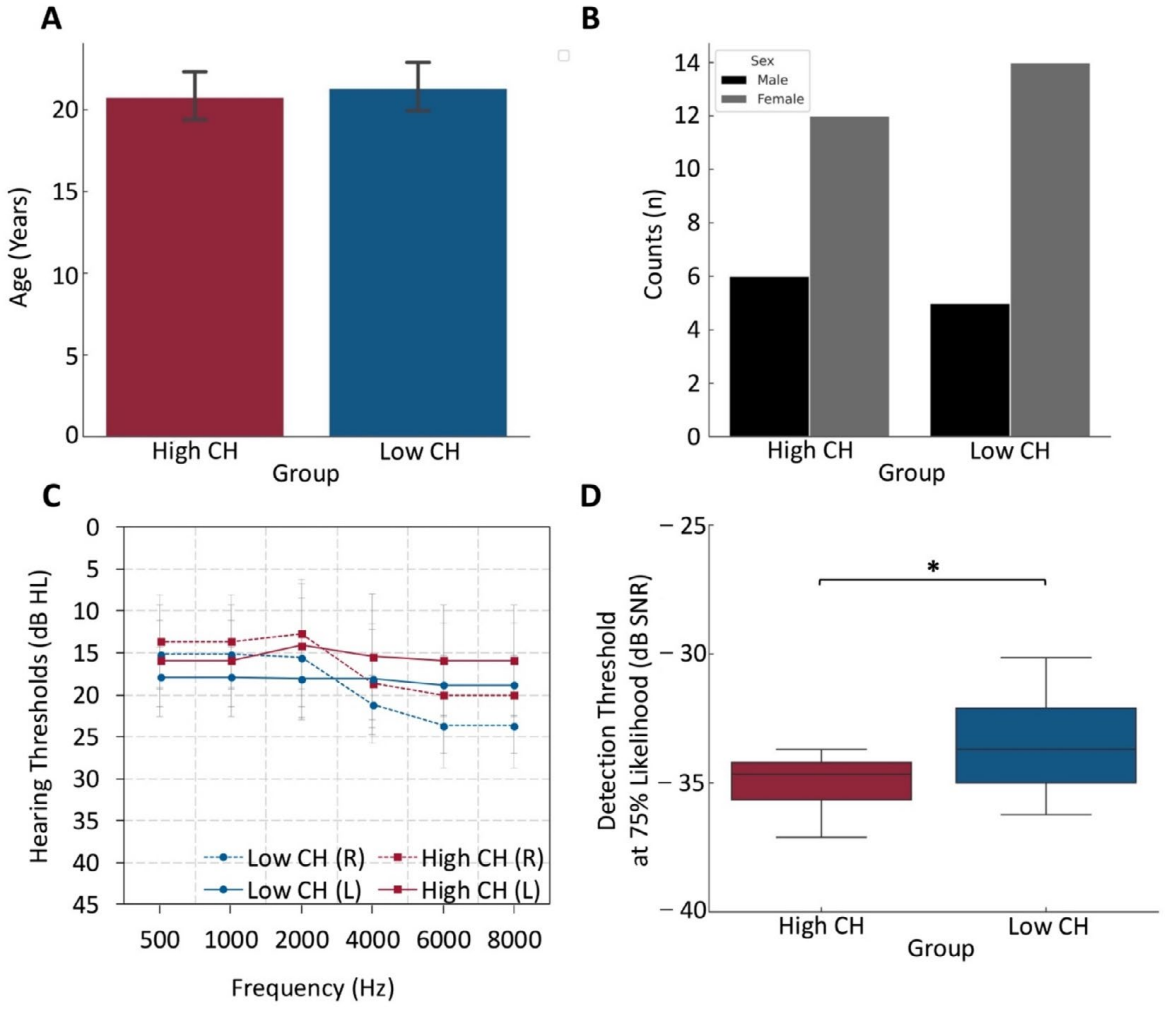
Demographical characteristics of high and low CH groups. Demographical summary showed no difference between the high and low perceivers in age (A), sex distribution (B) and hearing status (C) but lower detection thresholds in the high perceivers group (D). Data were presented as mean ± 95% CI. *p < 0.05.

The whole group and a categorization of the group into high CH perceivers (high perceivers) and low CH perceivers (low perceivers) based on a median split of the false alarm (FA) rate for the stimulus‐absent condition in the CH paradigm was used to answer the different research questions. The two groups were similar in age (*t*(35) = 0.489, *p* = 0.628, Cohen's *d* = 3.343), sex (*Χ* ^2^ = 0.218, *p* = 0.641) and hearing loss (*F*(5,175) = 1.581, *p* = 0.211, partial *η* ^2^ = 0.043) (Figure 1A–C). Furthermore, the detection threshold at 75% likelihood between the two groups (high perceivers = −34.96 ± 1.70; low perceivers = −33.09 ± 2.67) were compared using an independent *t*‐test. Although, the detection threshold for each person was used as their own internal control, there was an overall significant difference in detection threshold between the two groups (*t*(35) = 2.517, *p* = 0.017, Cohen's *d* = 2.250) (Figure 1D), suggesting that the high perceivers were possibly more sensitive to detecting external sensory information.

### 2.3 Stimuli and Experimental Design

All participants underwent the auditory CH and the local–global auditory oddball paradigms. The order of the two paradigms was fully randomized among participants. For the CH paradigm, the audio files and visual stimuli were produced in MATLAB. For the local–global paradigm, the audio files were produced in Adobe Audition. The experiments were presented through Matlab and Psychopy.

### 2.4 Experiment 1: Auditory CH Paradigm

This paradigm consisted of a behavioral and an electrophysiological session. The goal of the behavioral session was to determine the 75% likelihood threshold for the target auditory stimulus embedded in noise and cued using a visual stimulus. The QUEST maximum likelihood‐based procedure was used to estimate individual psychoacoustic thresholds for the CH paradigm. The QUEST algorithm was implemented in two 40‐trial long initially ascending interleaved staircases of step size determined by the QUEST program based on the participant responses. The target auditory stimulus was a 300 ms, 1 k Hz pure tone with a 10 ms rise‐fall time embedded in 40 dB SPL continuous background noise. A gray and black checkerboard with gray squares at 25% brightness served as a visual cue. Each trial in the behavioral session consisted of the 300 ms audio‐visual (target‐cue) stimulus embedded in continuous background noise and a 500–1000 ms inter‐trial interval. Participants were asked to indicate the presence or absence of the auditory stimulus concurrent with the visual cue as soon as possible using the left/right mouse click. The left/right click indicating “yes”/“no” was also randomized across subjects.

This session terminated, giving the 75% likelihood threshold of the amplitude of the tone embedded in noise. Additionally, individual psychometric curves were fitted to obtain 50% and 25% likelihood points of the amplitude of the sound in noise. The average thresholds at 75%, 50%, and 25% likelihoods between the two runs were considered for the electrophysiological session.

**FIGURE 2:**
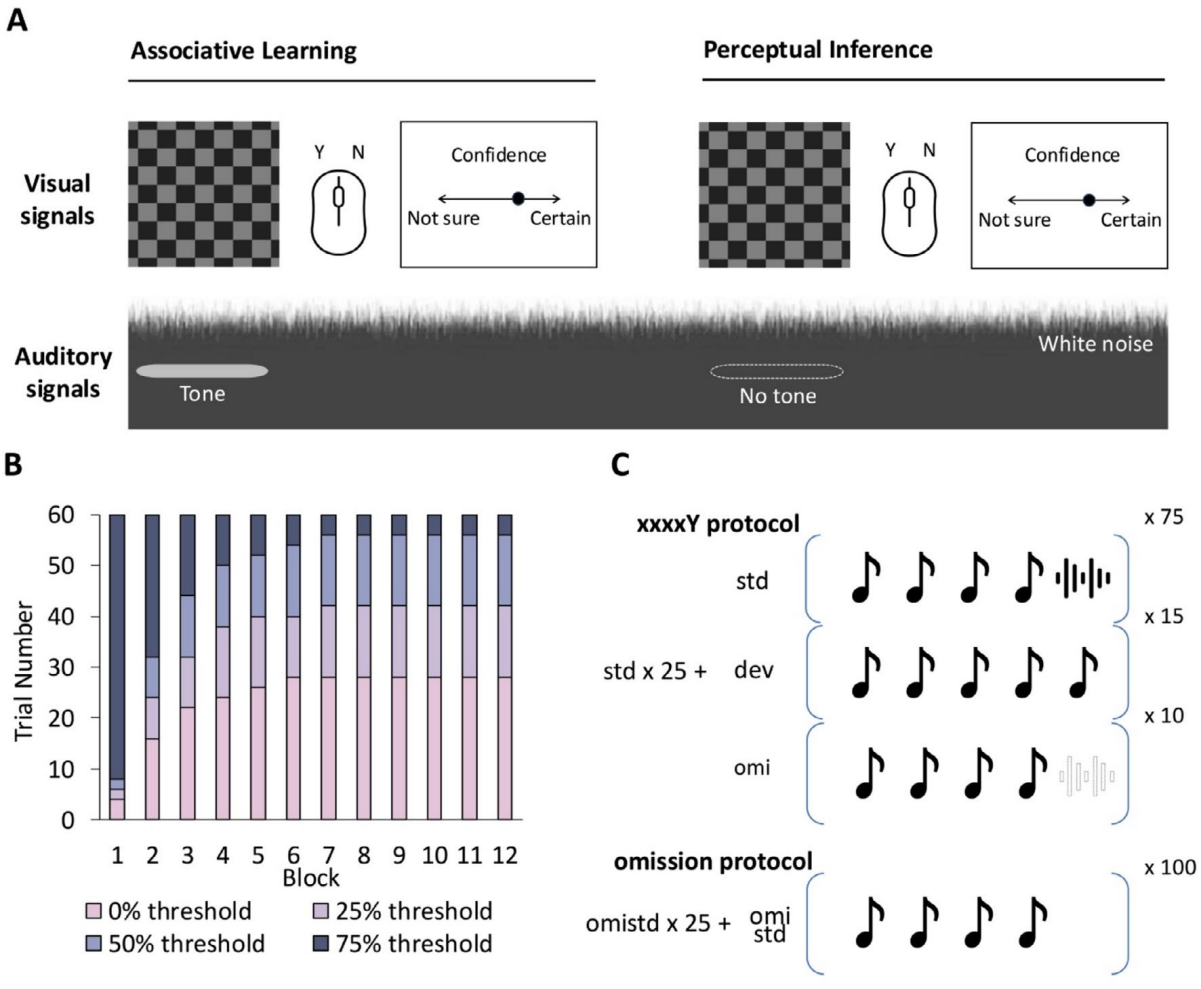
Study design for the auditory CH paradigm and oddball paradigm. (A) In the CH task, subjects were first implicitly taught the association between a visual checkerboard and a 1 k Hz tone in noise. During the perceptual inference phase, participants were required to indicate whether they heard a tone when a visual checkerboard was present by clicking the mouse. Participants were then asked to rate their confidence in this decision using a visual analogue scale from “Not sure” to “Very certain”. (B) Four stimulation conditions were estimated individually based on the psychometric curves of the tone detection threshold from the QUEST maximum‐based likelihood estimation test, and the proportion of four conditions was structured in a non‐linear manner. Threshold tones were more likely to be present in the earlier blocks, and absent tones were more likely to occur later, with systematically varied steps over 12 blocks of 60 trials. Subjects were divided into high or low perceivers based on the percentage of positive responses in the 0% threshold condition. (C) The oddball task consisted of three auditory stimulus sequences: Std.—sound sequence with four tones and a noise burst, dev—sound sequence with five monotonous stimuli, and omi—sound sequence with four tones but absent fifth stimulus. The xxxxY protocol consisted of seven blocks which started with 25 trials of std. sequences. In the following 100 trials, std. sequences were presented with 75% probability, the dev sequences with 15%, and the omi sequences with 10% probability. The omission protocol consisted of omistd—the omi sequence presented with 100% probability.

The electrophysiological session consisted of 12 blocks of 60 trials. Each trial started with a 300–500 ms fixation cross. This was succeeded by the 300 ms visual checkerboard with a total size of 1920 × 1080 pixels displayed on a 21 screen, resulting in a visual angle of around 33° presented concurrently with the auditory stimulus, followed by a 1000 ms period to allow for the development of the (ERP) to the multisensory stimulus. Participants were then required to indicate whether they heard a tone or not within a 1500 ms response window using a left/right mouse click and rate their confidence in this decision from “Not sure” to “Very certain” within 2000 ms (Figure 2A).

The distribution of the four stimulation conditions (75%, 50%, 25%, 0%) was structurally designed to first establish the latent association between the target tone and the visual cue and then test the perceptual decision with changing sensory uncertainty. The first two blocks overrepresented the 75% condition followed by a non‐linear decrease in the probability of presenting threshold‐level tones and an increase in the presentation of absent trials (Figure 2B).

The positive “yes” responses to 75%, 50%, and 25% were defined as Hits (HT) and negative “no” responses were defined as Misses (MS). In the 0% condition, the positive “yes” responses were defined as FA that corresponded to the CH, and the negative “no” responses were defined as Correct Rejection (CR). The percentage of hits, misses, FAs, and CRs after accounting for unresponsive trials was calculated for the 75%, 50%, 25%, and 0% (stimulus‐absent) conditions.

### 2.5 Experiment 2: Auditory Local–Global Oddball Paradigm

The second experiment conducted was a well‐established auditory local–global paradigm designed to elicit a PE based on changes in stimulus characteristics and probabilities. We used 500 Hz tones and broad‐band noise bursts, both of which were 50 ms long with a rise‐fall time of 7 ms. The stimuli were presented at 60 dB SPL. The paradigm consisted of three sound sequences–(i) standard: Four tones followed by a noise burst, (ii) deviant: Five tones, (iii) omission: Four tones with a missing fifth stimulus. These were presented in an oddball fashion in seven blocks. Each block started with 25 standards followed by the standard presented with 75% probability (std), deviant presented with 15% probability (dev) and the omission presented with 10% probability (omi). The sequences in the oddball presentation were pseudonymised to not present more than three standards consecutively. The omission sequence was also separately presented in a block by itself where it was 100% expected (omistd) (Figure 2C). These four conditions give rise to two types of PEs viz. (i) stimulus‐driven PE (sPE) = dev—std, a PE that is driven by a surprise in the fifth stimulus and (ii) context‐driven PE (cPE) = omi—omistd, a PE that is driven by the change in the probability of the exact same stimulus.

The oddball paradigm consisted of 8 blocks of 125 trials. Each trial started with a 1250 ms sound sequence followed by a random inter‐trial jitter between 700 and 1000 ms. The xxxxY protocol included 7 blocks, whereas the omission protocol had 1 block. The order of the two protocols was randomized across subjects. In the xxxxY protocol, each block started with 25 standard sequences and the following 100 trials included 75% standard trials (std), 15% deviant trials (dev) and 10% omission trials which were unexpected (omi). The omission protocol included 125 omission trials where all omission sequences were expected (omistd). During the whole experiment, participants were asked to maintain focus on a fixation cross at the centre of the screen.

### 2.6 EEG Data Collection and Pre‐Processing

The ERP data was collected using a 64‐channel cap configured as per the International 10–20 placement system. The data was sampled at 4096 Hz and recorded by the Bio Semi ActiveTwo system. Data was pre‐processed using MATLAB, EEGLAB v2021.1, and ERPLAB v8.20. The data was first examined for noisy channels due to sudden disconnection during the recording, bridging, etc. These, along with the unused channels, were first removed. The data was then down sampled to 500 Hz, re‐referenced to an average reference, and filtered using a band pass filter between 0.55 and 44 Hz. The data was further epoched based on the trial structure of individual paradigms. For the CH paradigm, the data was epoched from −300 to 1300 ms relative to the onset of the audio‐visual cue. For the Oddball paradigm, the data was epoched from −100 to 1850 ms relative to the onset of the first stimulus. The epoched data was then subjected to independent component analysis (ICA) to remove muscle artifacts, eye blinks, saccades, and other noise transients. The number of ICA components in both high and low perceivers were comparable for the CH condition (high perceivers: 50.06 ± 7.11, low perceivers: 48.95 ± 4.47, *t*(36) = 0.571, *p* = 0.572, Cohen's *d* = 0.187) and the oddball paradigm (high: 49.11 ± 3.10, low: 48.00 ± 2.36, *t*(36) = 1.231, *p* = 0.227, Cohen's *d* = 0.426). Any other artifacts from the epoched and ICA‐cleaned data were detected and deleted using a simple voltage threshold of ±90 μV and finally manually inspected to ensure that the data was intact. The channels initially removed were finally interpolated using a spherical interpolation algorithm in EEGLAB to ensure all participants had 64 channels.

The study takes a three‐step process to determine the relationship between different components of the predictive coding model to enable perception:

#### 2.6.1 Investigating the Role of Confidence in Building Priors Using Behavioral Data

The positive “yes” response to the 75%, 50%, and 25% tone thresholds was defined as Hit (HT) and the negative “no” response as Miss (MS), whereas in the 0% condition, the positive “yes” response to the 0% tone threshold was defined as FA that corresponded to the CH and the negative “no” as CR. For the response measure, the number of positive and negative responses was calculated for each of the stimulus conditions. The response rate of positive responses for each stimulus condition was defined as the ratio of the number of positive responses to the total number of trials corrected for trials with no responses. The response preference was defined as the subtraction of the negative response rate from the positive response rate.

For the confidence measure, mean confidence in positive and negative responses was calculated respectively for each stimulus condition. The relative confidence was calculated by subtracting the mean confidence in negative responses from that of the positive responses. The response rate, response preference (i.e., positive response rate—negative response rate), mean confidence rating, and relative confidence (i.e., confidence in positive response—confidence in negative response) were compared between the two groups and stimulus conditions (75%, 50%, 25%, 0%) using repeated measures ANOVA in which group (two groups) was the between‐subject factor and stimulus condition (four levels) was the repeated measure. Post hoc analysis was conducted using an independent samples *t*‐test corrected for multiple comparisons using a Benjamini‐Hochberg procedure with a FDR rate of 10%.

To investigate how the number of positive responses and relative confidence changed over the blocks and stimulus condition between the two groups, a linear mixed effect (LME) model was applied. Here, groups (two groups), blocks (12 levels) and stimulus conditions (4 levels) were used as the fixed factors, and subjects (37 subjects) were used as the random factor. The model was built in a hierarchical fashion by looking at different contrasts at different stages. If a significant groups × blocks × conditions effect was observed, a group × block LME was applied for each condition. A significant group × block effect for each condition was then subjected to post hoc analysis of group differences for each block using an independent *t*‐test. These tests were corrected for multiple comparisons using the Benjamini‐Hochberg FDR rate of 10%.

The relationship between relative confidence and response preference for the whole group at each stimulus condition was investigated using Pearson's correlation. This was corrected for multiple comparisons using a Benjamini‐Hochberg FDR rate of 10%. The steepness of the slope for the two groups was compared using a linear regression analysis which estimated the slopes for the high CH and low perceivers separately, followed by a difference using a *t*‐test.

The relationship between response preference and relative confidence for each condition with the value of the 75% perceptual threshold for the whole group was investigated using Pearson's correlation. This was corrected for multiple comparisons using a Benjamini‐Hochberg FDR rate of 10%. The steepness of the slope for the two groups was compared using a linear regression analysis which estimated the slopes for the high and low perceivers separately, followed by a difference using a *t*‐test.

#### 2.6.2 Investigating the Role of Priors in Building the Internal Model Using ERP Data

The neural signatures for the whole group and the group differences between the neural signatures were computed using non‐parametric cluster‐based permutation tests. Here, a dependent/independent samples *t*‐test was calculated for each channel‐timepoint sample between different conditions in the whole group or between the same measure in the two groups with the alpha of 0.05 as the cluster‐based threshold (two‐tailed) using FieldTrip. The cluster of neighboring channels for each channel was calculated using a standard template of the Biosemi 64 channel cap provided by FieldTrip. The minimum number of neighbouring channels to form a cluster was two. The resulting clusters were corrected for multiple comparisons using max‐T, which takes the sum of the t‐values within every cluster and compares them with the probability of clusters that were calculated using 1000 Monte‐Carlo permutations (Maris & Oostenveld, 2007). Clusters that were larger than 95% of the distribution were considered significant (*α* = 0.05, two‐tailed). The positive/negative cluster that showed the most significant difference between the two conditions or the two groups was selected.

We used the above cluster‐based permutation test to compare the trial‐averaged subject‐level evoked responses for the HT 75%, 50%, and 25% with the corresponding MS 75%, 50%, and 25% for the whole group and for the HT 75%, 50%, and 25% between the high and low perceivers. Notably, for the MS 75%, HT 25%, and FA conditions, all subjects inevitably had a significantly smaller number of trials for their paired conditions (i.e., HT75%, MS25% and CR) due to the experimental design. Therefore, for the HT75% versus MS75% comparison, we randomly sampled the 38 trials of HT75% trials (average number of MS75% trials for the whole group) for each person. For the HT25% versus MS25% comparison, we randomly sampled the 31 trials of MS25% trials (average number of HT25% trials for the whole group) for each person. For the FA versus CR comparison, we randomly sampled the 32 trials of CR trials (average number of FA trials for the whole group) for each person. Then, we compared the trial‐averaged ERPs between the two paired conditions by the cluster‐based permutation test. Both procedures were performed 100 times to procure a *t*‐test map. Those electrodes that showed significant results in greater than 80/100 comparisons were selected to be plotted.

For the between‐group comparison, We then compared the trial‐averaged FA with the CR condition. For comparison between the two groups, we randomly subsampled 50 trials (average number of FA trials in the high perceiver group) from the CR condition for each subject in the low perceivers. Then, we compared the trial‐averaged CR ERPs from the low perceivers with the trial‐averaged FA ERP from the high perceivers using the cluster‐based permutation test. Both procedures were performed 100 times to procure a *t*‐test map. Those electrodes that showed significant results in greater than 80/100 comparisons were selected to be plotted. The average amplitude for the significant channel‐time point cluster for each subject was correlated with the average confidence for the signal‐absent FA and CR for the high and low perceivers.

#### 2.6.3 Investigating the Role of PEs in Making Internal Models Using ERP Data

For the PE session, the same cluster‐based procedure was conducted for the comparison of trial‐averaged subject‐level evoked responses between omi and omistd, as well as dev and std. for the whole group. The amplitude over the significant channel‐timepoint cluster was averaged for each group (high and low perceivers) and each condition (std, dev, omi, omistd). A repeated‐measures ANOVA was performed with groups (high and low perceivers) as between subjects and conditions (std, dev; omi, omistd) as repeated measures. If there was a significant interaction effect, a univariate ANOVA was performed to reveal a significant group effect for each condition.

The stimulus‐driven PE (sPE) and context‐driven PE (cPE) were obtained by performing dev—std. and omi—omistd respectively from the averaged channel‐timepoint cluster. This was further subject to a repeated measures ANOVA with group (high and low perceivers) as between subjects and PE condition (sPE, cPE) as repeated measures. If a significant effect was obtained, a univariate ANOVA was performed to examine a group difference for each PE condition.

As an exploratory analysis, we obtained the subjective measure of past experience of perception of hallucinations. We asked the question “In the past, I have had the experience of hearing or seeing something then found that nothing was there” and asked them to draw a vertical line on a 10 cm visual analog scale going from “never” to “always”. We correlated this subjective hallucination score (SHS) with the FA rate using Pearson's correlation. We also performed a partial correlation between FA rate and SHS after correcting for the 75% detection threshold.

## 3 Results

### 3.1 Investigating the Role of Confidence in Building Priors

During the course of the 12 blocks, all participants made perceptual decisions about the auditory target when cued by the visual checkerboard. Based on previous literature, we hypothesized that confidence weights learning and aids in building strong internal models (investigated in 1.) Furthermore, we hypothesized that people who are more likely to perceive a false percept rely more on the internal model to make perceptual decisions when the auditory environment becomes more uncertain (investigated in 2. and 3).

#### 3.1.1 By Studying the Average Response Rate, Confidence, Response Preference, and Relative Confidence Over 12 Blocks

**FIGURE 3:**
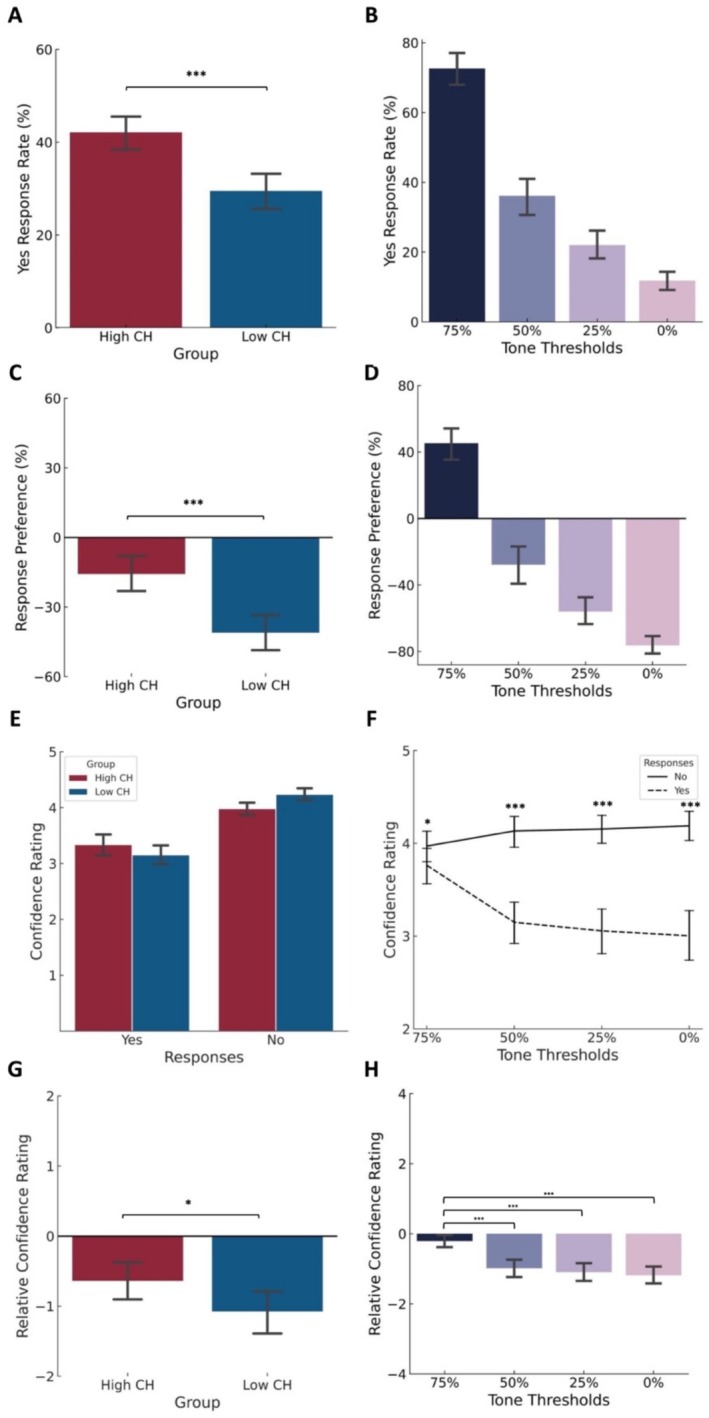
Behavioral results across the blocks in the CH task. Difference in positive response rate (A) between two groups and (B) across four stimulus conditions. Difference in response preference that is (positive response rate—negative response rate) (C) between two groups and (D) across four stimulus conditions. (E) The group by response difference in mean confidence ratings and (F) mean confidence ratings for positive and negative responses in four tone conditions. (G) Group difference of relative confidence rating (confidence in positive responses—confidence in negative responses). (H) The difference of relative confidence in four stimulus conditions. Data were present as mean ± 95% CI. *p < 0.05; **p < 0.01; ***p < 0.*001*.

Overall, we observed a significant main effect of group (*F*(1,35) = 19.734, *p* < 0.001, partial *η* ^2^ = 0.361) (Figure 3A) and stimulus condition (*F*(3,105) = 314.727, *p* < 0.001, partial *η* ^2^ = 0.900) (Figure 3B) for the positive response rate, with the high perceivers having a significantly higher positive response rate compared to low perceivers, and the positive response rate decreasing with increasing stimulus uncertainty. We observed a similar trend for the response preference rate (group: *F*(1,35) = 19.734, *p* < 0.001, partial *η* ^2^ = 0.361, condition: *F*(3,105) = 314.727, *p* < 0.001, partial *η* ^2^ = 0.900) (Figure 3C,D). No significant group × condition interaction was observed either for the response rate (*F*(1,105) = 2.490, *p* = 0.091, partial *η* ^2^ = 0.066) nor for the response preference (*F*(1,105) = 2.490, *p* = 0.091, partial *η* ^2^ = 0.066). The pairwise comparison for conditions is provided in Table 1.

**TABLE 1:** Pairwise comparisons of ANOVAs involving contrasts of response rates, response preference, mean confidence and relative confidence.

Pairwise comparisons	Mean difference, *p*	Confidence intervals
Main effect of condition while comparing response rates (Figure 3B)		
75% versus 50%	MD = 0.364, *p* < 0.001	[0.327, 0.400]
75% versus 25%	MD = 0.504, *p* < 0.001	[0.458, 0.551]
75% versus 0%	MD = 0.607, *p* < 0.001	[0.553, 0.662]
50% versus 25%	MD = 0.141, *p* < 0.001	[0.104, 0.177]
50% versus 0%	MD = 0.244, *p* < 0.001	[0.192, 0.295]
25% versus 0%	MD = 0.103, *p* < 0.001	[0.078, 0.127]
Main effect of condition while comparing response preference (Figure 3D)		
75% versus 50%	MD = 0.728, *p* < 0.001	[0.655, 0.800]
75% versus 25%	MD = 1.009, *p* < 0.001	[0.916, 1.102]
75% versus 0%	MD = 1.215, *p* < 0.001	[1.106, 1.323]
50% versus 25%	MD = 0.281, *p* < 0.001	[0.209, 0.354]
50% versus 0%	MD = 0.487, *p* < 0.001	[0.383, 0.591]
25% versus 0%	MD = 0.206, *p* < 0.001	[0.156, 0.255]
Main effect of condition while comparing confidence to reporting yes/no (Figure 3F)		
75% versus 50%	MD = 0.224, *p* < 0.001	[0.102, 0.347]
75% versus 25%	MD = 0.267, *p* < 0.001	[0.119, 0.414]
75% versus 0%	MD = 0.272, *p* < 0.001	[0.096, 0.449]
50% versus 25%	MD = 0.042, *p* = 0.652	[−0.029, 0.114]
50% versus 0%	MD = 0.048, *p* = 1.00	[−0.063, 0.159]
25% versus 0%	MD = 0.006, *p* = 1.00	[−0.066, 0.078]
Main effect of condition while comparing relative confidence (Figure 3H)		
75% versus 50%	MD = 0.772, *p* < 0.001	[0.561, 0.983]
75% versus 25%	MD = 0.889, *p* < 0.001	[0.584, 1.193]
75% versus 0%	MD = 0.977, *p* < 0.001	[0.621, 1.332]
50% versus 25%	MD = 0.116, *p* = 0.343	[−0.049, 0.282]
50% versus 0%	MD = 0.205, *p* = 0.114	[−0.028, 0.437]
25% versus 0%	MD = 0.088, *p* = 0.550	[−0.054, 0.231]

Although we did not observe a 3‐way interaction of group × condition × response (*F*(3,105) = 2.041, *p* = 0.113, partial *η* ^2^ = 0.055), we observed that irrespective of the stimulus condition the low perceivers showed a trend towards increased confidence in reporting the absence of the target compared to the high perceivers (significant group × response: *F*(1,35) = 4.382, *p* = 0.044 partial *η* ^2^ = 0.111, with post hoc pairwise comparisons yes: *F*(1,35) = 0.698, *p* = 0.409, partial *η* ^2^ = 0.020; no: *F*(1,35) = 2.696, *p* = 0.110, partial *η* ^2^ = 0.072) (Figure 3E). We also observed that all people were more confident in reporting the absence of the auditory target compared to its presence at all threshold levels (significant response × condition: *F*(3,105) = 51.343, *p* < 0.001, partial *η* ^2^ = 0.595 with post hoc pairwise comparisons 75%: *F*(1,35) = 5.281, *p* = 0.028, partial *η* ^2^ = 0.131; 50%: *F*(1,35) = 67.091, *p* < 0.001, partial *η* ^2^ = 0.657; 25%: *F*(1,35) = 72.171, *p* < 0.001, partial *η* ^2^ = 0.673; 0%: *F*(1,35) = 86.986, *p* < 0.001, partial *η* ^2^ = 0.713) (Figure 3F). We did not observe a significant group × condition interaction (*F*(3,105) = 1.516, *p* = 0.215, partial *η* ^2^ = 0.042). We also observed a main effect of response (*F*(1,35) = 69.773, *p* < 0.001, partial *η* ^2^ = 0.666) and a main effect of condition (*F*(3,105) = 17.297, *p* < 0.001, partial *η* ^2^ = 0.331) but no main effect of group (*F*(1,35) = 0.56, *p* = 0.814, partial *η* ^2^ = 0.002). The pairwise comparison for conditions is provided in Table 1.

Although taking the relative confidence of reporting yes versus no to the presence of the target, we observed no significant group × condition interaction (*F*(3,105) = 2.073, *p* = 0.147, partial *η* ^2^ = 0.056). However, we observed that irrespective of the stimulus condition, the low perceivers were more confident in reporting the absence of the target compared to the high perceivers (main effect of group: *F*(1,35) = 4.473, *p* = 0.042, partial *η* ^2^ = 0.113) (Figure 3G). Furthermore, we observed that at 75% likelihood, when all participants could reliably perceive the target, the relative confidence of yes versus no was close to zero. As the uncertainty of the target increased, the relative confidence of yes versus no became < 0 that is people were more confident in reporting the absence of the target main effect of condition: *F*(3,105) = 51.444, *p* < 0.001, partial *η* ^2^ = 0.595 with post hoc comparisons given in Table 1 (Figure 3H).

#### 3.1.2 By Studying the Number of Positive Responses and Relative Confidence Over 12 Blocks

However, when we looked through the course of the 12 blocks and compared how high and low perceivers build internal models using the linear mixed model, we observed a 3‐way interaction between group × response × condition for the number of responses (*F*(95,1188.071) = 136.918, *p* < 0.001, partial *η* ^2^ = 0.916) and the relative confidence (*F*(95,1178.963) = 4.768, *p* < 0.001, partial *η* ^2^ = 0.277).

**FIGURE 4:**
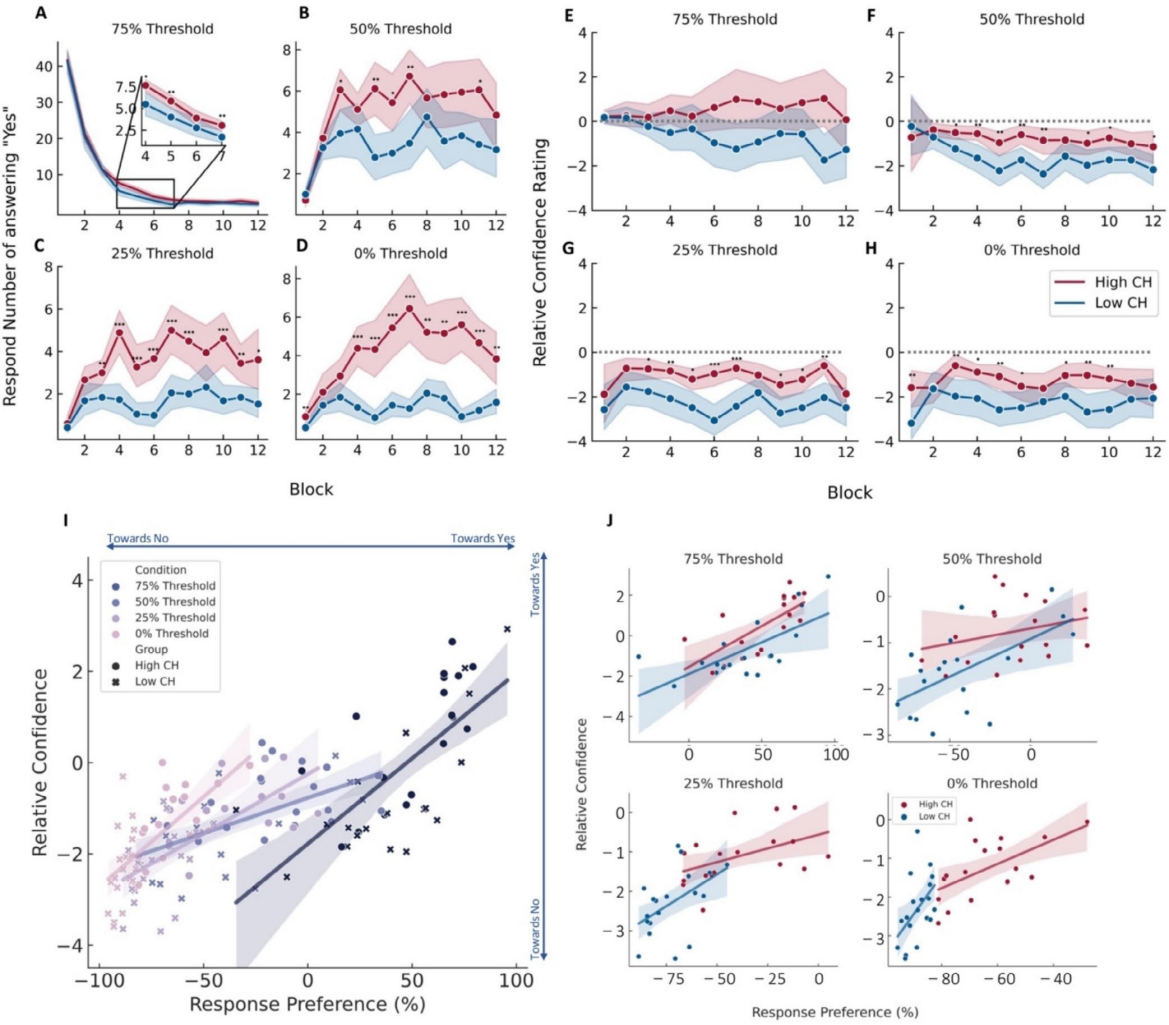
Behavioural results over the blocks in the CH task. The group difference in the number of positive responses per block in the (A) 75%, (B) 50% (C) 25% and (D) 0% tone thresholds. The group difference of relative confidence rating per block in the (E) 75%, (F) 50%, (G) 25% and (H) 0% tone thresholds. Gray dashed lines represent the equalized confidence rating between positive and negative responses. The correlation between response preference and relative confidence for (I) all subjects and (J) for each group in each tone threshold condition.

When the signal was detectable (i.e., at 75% likelihood) there was a significant difference in the number of responses (group × block: *F*(23, 313.17) = 373.014, *p* < 0.001, partial *η* ^2^ = 0.964) (Figure 4A) but not the relative confidence (group × block: *F*(23, 320.432) = 1.498, *p* = 0.068, partial *η* ^2^ = 0.097) (Figure 4E) between the two groups in the first few blocks where the number of 75% likelihood stimuli was the highest. The relative confidence was close to 0, suggesting that both groups relied on the signal itself to respond.

As the uncertainty of the signal increased, we observed the same pattern as the average in both the number of responses and relative confidence. We also observed the additional dynamic that the number of responses (group × block: 50%: *F*(23, 324.778) = 6.752, *p* < 0.001, partial *η* ^2^ = 0.323; 25%: *F*(23,309.408) = 6.797, *p* < 0.001, partial *η* ^2^ = 0.336; 0%: *F*(23, 302.257) = 8.615, *p* < 0.001, partial *η* ^2^ = 0.396; with pairwise comparisons showing significant results with *p* < 0.05 shown in Table 2) (Figure 4B–D) and the relative confidence (group × block: 50%: *F*(23, 298.226) = 2.381, *p* < 0.001, partial *η* ^2^ = 0.155; 25%: *F*(23, 294.973) = 2.615, *p* < 0.001, partial *η* ^2^ = 0.169; 0%: *F*(23, 299.488) = 1.892, *p* = 0.009, partial *η* ^2^ = 0.127; with pairwise comparisons showing significant results with *p* < 0.05 shown in Table 3) (Figure 4F–H) in the high perceivers was higher than those of the low perceivers. This suggested that as the target became increasingly less likely to be present, the low perceivers were more confident and accurate in reporting this information than the high perceivers. That is, low perceivers continued to rely on the sensory information even when the target became increasingly uncertain.

**TABLE 2:** Difference between response number between the two groups at each stimulus condition for each block.

Pairwise comparisons	Mean difference, *p*	Confidence intervals
75% threshold (Figure 4A)		
Block 1	MD = 0.456, *p* = 0.830	[−3.820, 4.732]
Block 2	MD = 1.020, *p* = 0.593	[−2.825, 4.866]
Block 3	MD = 0.184, *p* = 0.869	[−2.069, 2.438]
Block 4	MD = 2.137, *p* = 0.010	[0.537, 3.738]
Block 5	MD = 1.833, *p* = 0.009	[0.495, 3.171]
Block 6	MD = 1.099, *p* = 0.102	[−0.231, 2.430]
Block 7	MD = 1.319, *p* = 0.006	[0.411, 2.226]
Block 8	MD = 0.351, *p* = 0.413	[−0.509, 1.211]
Block 9	MD = 0.611, *p* = 0.145	[−0.220, 1.443]
Block 10	MD = 0.178, *p* = 0.680	[−0.692, 1.049]
Block 11	MD = 0.827, *p* = 0.079	[−0.100, 1.755]
Block 12	MD = 0.433, *p* = 0.366	[−0.527, 1.392]
50% Threshold (Figure 4B)		
Block 1	MD = −0.278, *p* = 0.290	[−0.802, 0.247]
Block 2	MD = 0.459, *p* = 0.335	[−0.494, 1.412]
Block 3	MD = 2.108, *p* = 0.013	[0.481, 3.736]
Block 4	MD = 0.953, *p* = 0.249	[−0.699, 2.606]
Block 5	MD = 3.322, *p* < 0.001	[1.512, 5.131]
Block 6	MD = 2.444, *p* = 0.010	[0.623, 4.266]
Block 7	MD = 3.249, *p* = 0.003	[1.166, 5.331]
Block 8	MD = 0.930, *p* = 0.388	[−1.229, 3.088]
Block 9	MD = 2.254, *p* = 0.053	[−0.034, 4.543]
Block 10	MD = 2.102, *p* = 0.048	[0.017, 4.188]
Block 11	MD = 2.635, *p* = 0.014	[0.561, 4.708]
Block 12	MD = 1.675, *p* = 0.131	[−0.522, 3.883]
25% Threshold (Figure 4C)		
Block 1	MD = 0.190, *p* = 0.411	[−0.274, 0.654]
Block 2	MD = 0.982, *p* = 0.071	[−0.089, 2.054]
Block 3	MD = 1.158, *p* = 0.009	[0.309, 0.2006]
Block 4	MD = 3.152, *p* < 0.001	[1.791, 4.513]
Block 5	MD = 2.225, *p* < 0.001	[1.082, 3.368]
Block 6	MD = 2.667, *p* < 0.001	[1.549, 3.784]
Block 7	MD = 2.947, *p* < 0.001	[1.438, 4.457]
Block 8	MD = 2.500, *p* < 0.001	[1.191, 3.809]
Block 9	MD = 1.629, *p* = 0.075	[−0.174, 3.431]
Block 10	MD = 2.927, *p* < 0.001	[1.445, 4.409]
Block 11	MD = 1.602, *p* = 0.006	[0.502, 2.702]
Block 12	MD = 2.085, *p* = 0.015	[0.437, 3.732]
0% Threshold (Figure 4D)		
Block 1	MD = 0.570, *p* < 0.001	[0.176, 0.964]
Block 2	MD = 0.690, *p* = 0.195	[−0.371, 1.751]
Block 3	MD = 1.102, *p* = 0.064	[−0.068, 2.272]
Block 4	MD = 3.073, *p* < 0.001	[1.835, 4.311]
Block 5	MD = 3.544, *p* < 0.001	[1.935, 5.153]
Block 6	MD = 4.023, *p* < 0.001	[2.271, 5.776]
Block 7	MD = 5.181, *p* < 0.001	[3.313, 7.050]
Block 8	MD = 3.170, *p* < 0.001	[1.458, 4.882]
Block 9	MD = 3.377, *p* = 0.001	[1.443, 5.311]
Block 10	MD = 4.769, *p* < 0.001	[3.226, 6.312]
Block 11	MD = 3.509, *p* < 0.001	[2.079, 4.939]
Block 12	MD = 2.254, *p* = 0.007	[0.662, 3.847]

**TABLE 3:** Difference between relative confidence between the two groups at each stimulus condition for each block.

Pairwise comparisons	Mean difference, *p*	Confidence intervals
75% threshold (Figure 4E)		
Block 1	MD = 0.048, *p* = 0.814	[−0.367, 0.464]
Block 2	MD = 0.101, *p* = 0.794	[−0.676 0.877]
Block 3	MD = 0.412, *p* 0.330	[−0.434, 1.258]
Block 4	MD = 0.987, *p* = 0.096	[−0.183, 2.158]
Block 5	MD = 0.564, *p* = 0.416	[−0.827, 1.955]
Block 6	MD = 1.586, *p* = 0.057	[−0.049, 3.221]
Block 7	MD = 2.230, *p* = 0.021	[0.354, 4.106]
Block 8	MD = 1.809, *p* = 0.037	[0.118, 3.500]
Block 9	MD = 1.118, *p* = 0.198	[−0.613, 2.851]
Block 10	MD = 1.420, *p* = 0.103	[−0.301, 3.141]
Block 11	MD = 2.762, *p* = 0.004	[0.930, 4.595]
Block 12	MD = 1.355, *p* = 0.186	[−0.682, 3.393]
50% Threshold (Figure 4F)		
Block 1	MD = −0.492, *p* = 0.677	[−2.867, 1.883]
Block 2	MD = 0.330, *p* = 0.281	[−0.282, 0.942]
Block 3	MD = 0.725, *p* = 0.037	[0.046, 1.403]
Block 4	MD = 1.095, *p* = 0.005	[0.346, 1.844]
Block 5	MD = 1.258, *p* = 0.010	[0.322, 2.193]
Block 6	MD = 1.135, *p* = 0.004	[0.397, 1.873]
Block 7	MD = 1.511, *p* = 0.002	[0.620, 2.402]
Block 8	MD = 0.726, *p* = 0.122	[−0.204, 1.656]
Block 9	MD = 0.987, *p* = 0.049	[0.004, 1.970]
Block 10	MD = 0.991, *p* = 0.020	[0.164, 1.819]
Block 11	MD = 0.730, *p* = 0.077	[−0.084, 1.543]
Block 12	MD = 1.035, *p* = 0.055	[−0.023, 2.097]
25% Threshold (Figure 4G)		
Block 1	MD = 0.685, *p* = 0.430	[−1.057, 2.426]
Block 2	MD = 0.844, *p* = 0.093	[−0.149, 1.837]
Block 3	MD = 1.012, *p* = 0.028	[0.116, 1.908]
Block 4	MD = 1.239, *p* = 0.007	[0.356, 2.121]
Block 5	MD = 1.276, *p* = 0.012	[0.297, 2.255]
Block 6	MD = 2.106, *p* < 0.001	[1.198, 3.015]
Block 7	MD = 1.712, *p* < 0.001	[0.816, 2.609]
Block 8	MD = 0.795, *p* = 0.109	[−0.187, 1.777]
Block 9	MD = 1.273, *p* = 0.020	[0.216, 2.331]
Block 10	MD = 1.265, *p* = 0.011	[0.309, 2.221]
Block 11	MD = 1.443, *p* = 0.002	[0.583, 2.302]
Block 12	MD = 0.623, *p* = 0.306	[−0.593, 1.840]
0% Threshold (Figure 4H)		
Block 1	MD = 1.599, *p* = 0.010	[0.399, 2.799]
Block 2	MD = 0.048, *p* = 0.932	[−1.081, 1.176]
Block 3	MD = 1.380, *p* = 0.006	[0.416, 2.345]
Block 4	MD = 1.186, *p* = 0.021	[0.190, 2.183]
Block 5	MD = 1.494, *p* = 0.006	[0.459, 2.530]
Block 6	MD = 0.959, *p* = 0.059	[−0.040, 1.955]
Block 7	MD = 0.581, *p* = 0.290	[−0.517, 1.678]
Block 8	MD = 0.955, *p* = 0.032	[0.085, 1.825]
Block 9	MD = 1.653, *p* = 0.002	[0.657, 2.649]
Block 10	MD = 1.374, *p* = 0.010	[0.343, 2.406]
Block 11	MD = 0.720, *p* = 0.208	[−0.419, 1.860]
Block 12	MD = 0.500, *p* = 0.434	[−0.780, 1.781]

#### 3.1.3 By Studying the Relationship Between Response Preference, Relative Confidence, and Perceptual Threshold

Studying the relationship between relative confidence and response preference, we observed a strong positive relationship at every threshold hold level (75%: *R* = 0.743, *p* < 0.001, 50%: *R* = 0.623, *p* < 0.001, 25%: *R* = 0.735, *p* < 0.001, 0%: *R* = 0.686, *p* < 0.001) (Figure 4I). At the 75% threshold level, we observed that most high perceivers showed high confidence and a high response rate in perceiving the target stimulus, and most low perceivers showed low confidence and a low response rate. Furthermore, the strong positive relationship at every threshold suggested the involvement of confidence in building the internal model when the signal was detectable, and the involvement of confidence in making perceptual decisions when the stimulus became uncertain.

Comparing this relationship between the two groups, although we did not observe a difference in the steepness of the slope between the two groups, we observed that the distinction between the two groups increased. From having a similar relationship between the relative confidence and response preference at a 75% threshold (high perceivers: *F*(1,16) = 15.821, *p* = 0.001, *R* ^2^ = 0.497; low perceivers: *F*(1,17) = 15.016, *p* = 0.001, *R* ^2^ = 0.469; standardized *β* = 0.182, *t* = 0.685, *p* = 0.498) (Figure 4J), the groups gradually dissociated into distinct clusters in the stimulus‐absent condition, with high perceivers still having relatively higher confidence and higher response preference rates than low perceivers (0% high perceivers: *F*(1,16) = 8.288, *p* = 0.011, *R* ^2^ = 0.341; low perceivers: *F*(1,17) = 5.330 *p* = 0.034, *R* ^2^ = 0.239; standardized *β* = 2.425, *t* = 1.637, *p* = 0.111) (Figure 4J).

Furthermore, we also observed that when the stimulus was at 50% threshold that is equally probable and improbable to be perceived, the high perceivers showed no significant relationship between relative confidence and response (50% high perceivers: *F*(1,16) = 1.130, *p* = 0.275, *R* ^2^ = 0.074; low perceivers: *F*(1,17) = 9.152, *p* = 0.008, *R* ^2^ = 0.350; standardized *β* = 0.220, *t* = 1.217, *p* = 0.232) (Figure 4J). When the stimulus was more unlikely to be detected that is at 25% threshold, this relationship started to strengthen again (25% high perceivers: *F*(1,16) = 4.484, *p* = 0.050, *R* ^2^ = 0.219; low perceivers: *F*(1,17) = 5.326 *p* = 0.034, *R* ^2^ = 0.239; standardized *β* = 0.481, *t* = 1.239, *p* = 0.224) (Figure 4J). However, for the low perceivers, this relationship lasted throughout.

This suggests that subjective confidence shapes the way people learn in a multisensory environment. Furthermore, it was also evident that in the high perceivers, as the likelihood of the target became more uncertain, the dependence on the stimulus itself may be absent and the confidence to make a perceptual inference was dependent on their internal model. Those who were less likely to perceive a false percept, that is the low perceivers, were more likely to depend on the stimulus.

**FIGURE 5:**
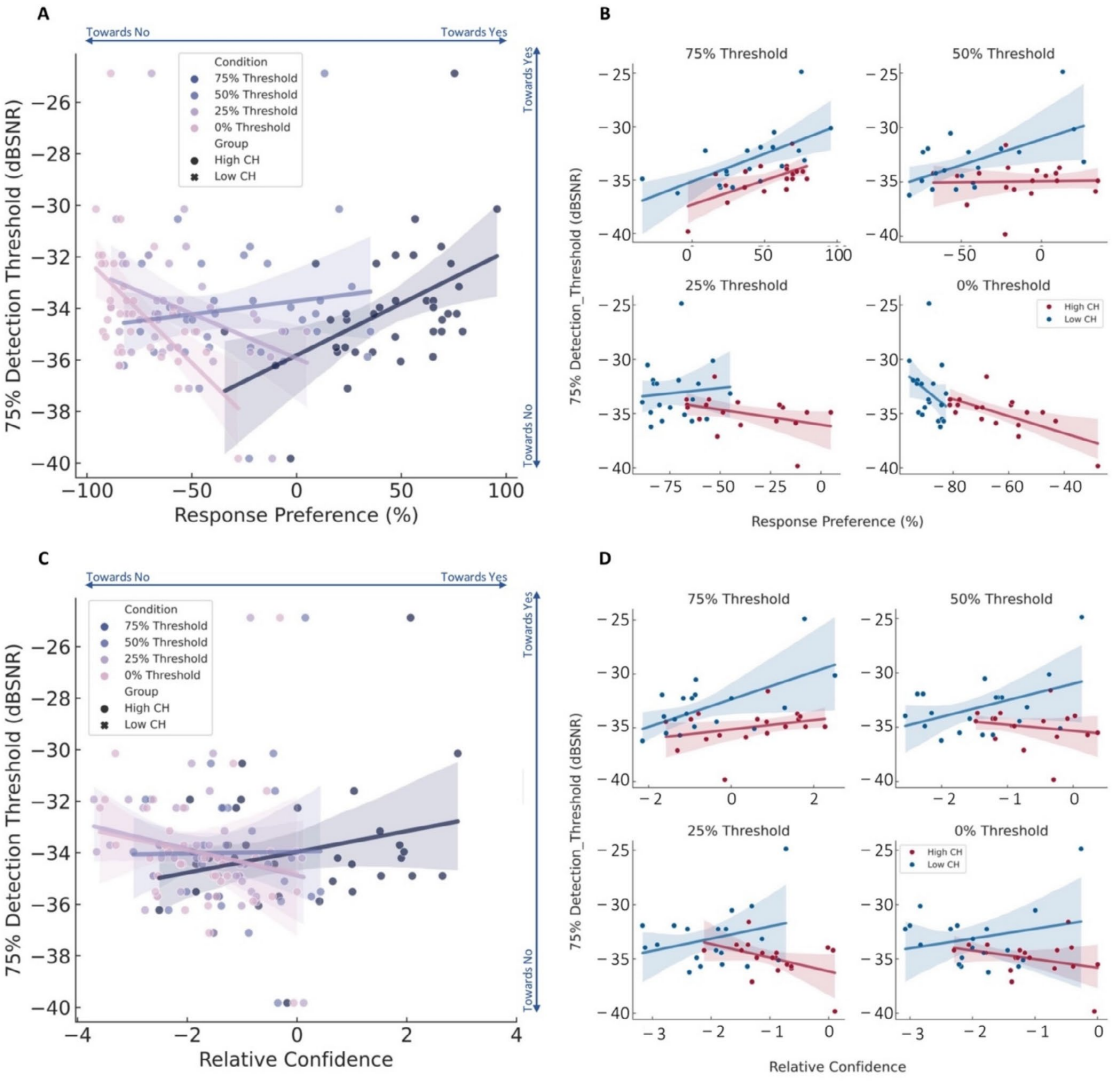
The correlation between behavioural results and 75% detection thresholds in the CH task. The correlation of 75% detection thresholds with response preference (A) and relative confidence (C) for all subjects. The correlation of 75% detection thresholds with response preference (B) and relative confidence (D) for each group in each tone threshold condition.

This is further established by the relationship between the perceptual threshold and response preference. As the stimulus gets increasingly uncertain, the relationship between the response preference and the 75% detection threshold changes direction. When the signal is detectable, we observe a positive relationship between the threshold and preference to say “yes” to the stimulus (75%: *R* = 0.479, *p* = 0.003, 50%: *R* = 0.144, *p* = 0.396) (Figure 5A), meaning that the more detectable the stimulus is, the more people are likely to report the presence of the stimulus. This is the case for both the high and low perceivers, and both groups depend on the stimulus itself (75% threshold high perceivers: *F*(1,16) = 11.176, *p* = 0.004, *R* ^2^ = 0.411; 75% threshold low perceivers: *F*(1,17) = 10.830, *p* = 0.004, *R* ^2^ = 0.389; standardized *β* = 0.454, *t* = 1.761, *p* = 0.087; 50% threshold high perceivers: *F*(1,16) = 0.016, *p* = 0.902, *R* ^2^ = 0.001; 50% threshold low perceivers: *F*(1,17) = 8.596, *p* = 0.009, *R* ^2^ = 0.336; standardized *β* = 0.807, *t* = 4.132, *p* < 0.001) (Figure 5B). As the signal gets increasingly less certain, we observe a negative relationship between the 75% perceptual threshold and the likelihood to say “yes” to the presence of the stimulus, meaning that as the threshold becomes lower (25%: *R* = −0.360, *p* = 0.029, 0%: *R* = −0.546, *p* < 0.001) (Figure 5A), people are more likely to say “yes” to the presence of the stimulus, even when it may be absent. We see this relationship particularly with the high perceiver group and not the low perceiver group, telling us that the high perceiver group may be more reliant on the internal model rather than the stimulus itself in the case when the stimulus is uncertain (25% threshold high perceivers: *F*(1,16) = 2.700, *p* = 0.120, *R* ^2^ = 0.144; 25% threshold low perceivers: *F*(1,17) = 0.142, *p* = 0.711, *R* ^2^ = 0.008; standardized *β* = 0.911, *t* = 1.313, *p* = 0.198; 0% threshold high perceivers: *F*(1,16) = 13.507, *p* = 0.002, *R* ^2^ = 0.458; 0% threshold low perceivers: *F*(1,17) = 1.915, *p* = 0.184, *R* ^2^ = 0.101; standardized *β* = 2.386, *t* = 1.059, *p* = 0.297) (Figure 5B).

This relationship is not present between the 75% detection threshold and the relative confidence (75%: *R* = 0.256, *p* = 0.127; high perceivers *F*(1,16) = 1.606, *p* = 0.223, *R* ^2^ = 0.091; low perceivers: *F*(1,17) = 9.753, *p* = 0.006, *R* ^2^ = 0.365; standardized *β* = −0.294, *t* = −1.552, *p* = 0.130; 50%: *R* = 0.013, *p* = 0.937; high perceivers: *F*(1,16) = 0.599, *p* = 0.450, *R* ^2^ = 0.036; low perceivers: *F*(1,17) = 4.100, *p* = 0.059, *R* ^2^ = 0.194; standardized *β* = −0.448, *t* = −1.875, *p* = 0.070; 25%: *R* = −0.207, *p* = 0.219; high perceivers: *F*(1,16) = 3.802, *p* = 0.069, *R* ^2^ = 0.192; low perceivers: *F*(1,17) = 1.662, *p* = 0.215, *R* ^2^ = 0.089; standardized *β* = −0.619, *t* = −2.071, *p* = 0.056; 0%: *R* = −0.179, *p* = 0.289; high perceivers: *F*(1,16) = 1.739, *p* = 0.206, *R* ^2^ = 0.098; low perceivers: *F*(1,17) = 1.082, *p* = 0.313, *R* ^2^ = 0.060; standardized *β* = −0.501, *t* = −1.557, *p* = 0.129) (Figure 5C,D).

##### 3.1.3.1 The Role of Priors in Making Internal Models

**FIGURE 6:**
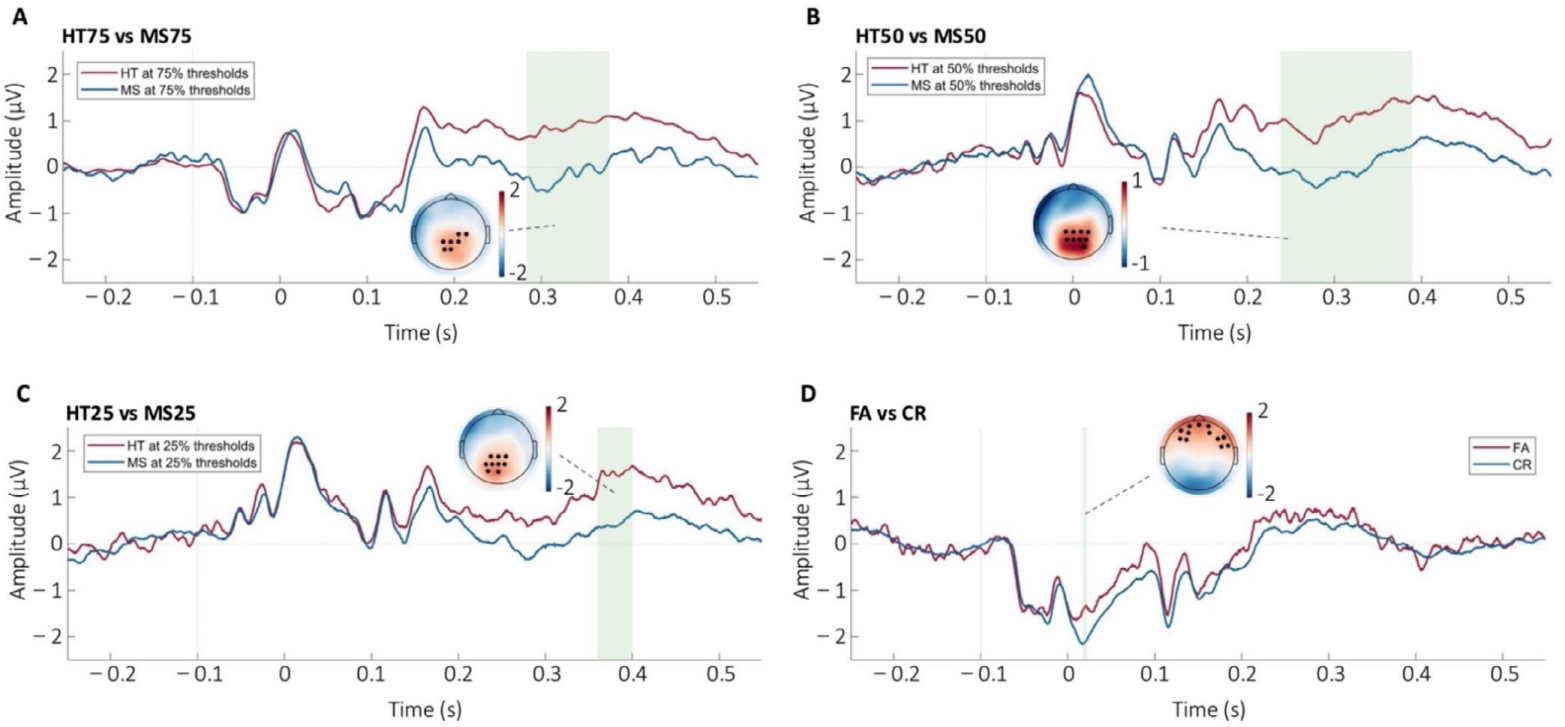
Within‐subject Neural response at different threshold conditions in the CH task. The ERPs were grand averaged over all subjects in each condition across the channels in black. The topographical plot represented the condition difference. The mean amplitude was averaged across the significant channels and timepoints (regions in green) between the positive response and negative response for each threshold condition. (A) shows the ERP and scalp plot of the Hit (HT) condition and the Miss (MS) condition at the 75% threshold condition, (B) shows the ERP and scalp plot of HT condition and MS condition at the 50% threshold condition (C) shows the ERP and scalp plot of HT condition and MS condition at the 25% threshold condition (D) shows the ERP and scalp plot of the false alarm (FA) condition and the correct rejection (CR) condition at the 0% threshold condition.

To further provide evidence for the above statement, we examined the electrophysiological responses of the CH paradigm for the whole group. Comparing the hits and misses at each threshold condition for the whole group, we observed a significant increase in the amplitude in a cluster of central‐parietal electrodes for the 75% (TOI: 766–956 ms, ROI: C2, C4, CPz, CP1, CP2, CP4, Pz, P1, cluster‐based tmax = 5954.9 *p* < 0.001), 50% (TOI: 676–978 ms, ROI: CPz, CP1, CP2, CP4, Pz, P1, P2, P4, PO4, cluster‐based tmax = 2160.6, *p* < 0.001) and 25% TOI: 924–1002 ms, ROI: CPz, CP1, CP2, Pz, P1, P2, P3, POz, PO3, cluster‐based tmax = 2644.3, *p* = 0.004 threshold conditions and a significant increase in a frontal cluster of electrodes for the stimulus absent condition (TOI: 234–244, ROI: Fpz, Fp1, Fp2, AF3, AF4, AF7, F3, F5, F6, F8, FT8, FC6, cluster‐based tmax = 4430.3, *p* < 0.001) (Figure 6A–D).

**FIGURE 7:**
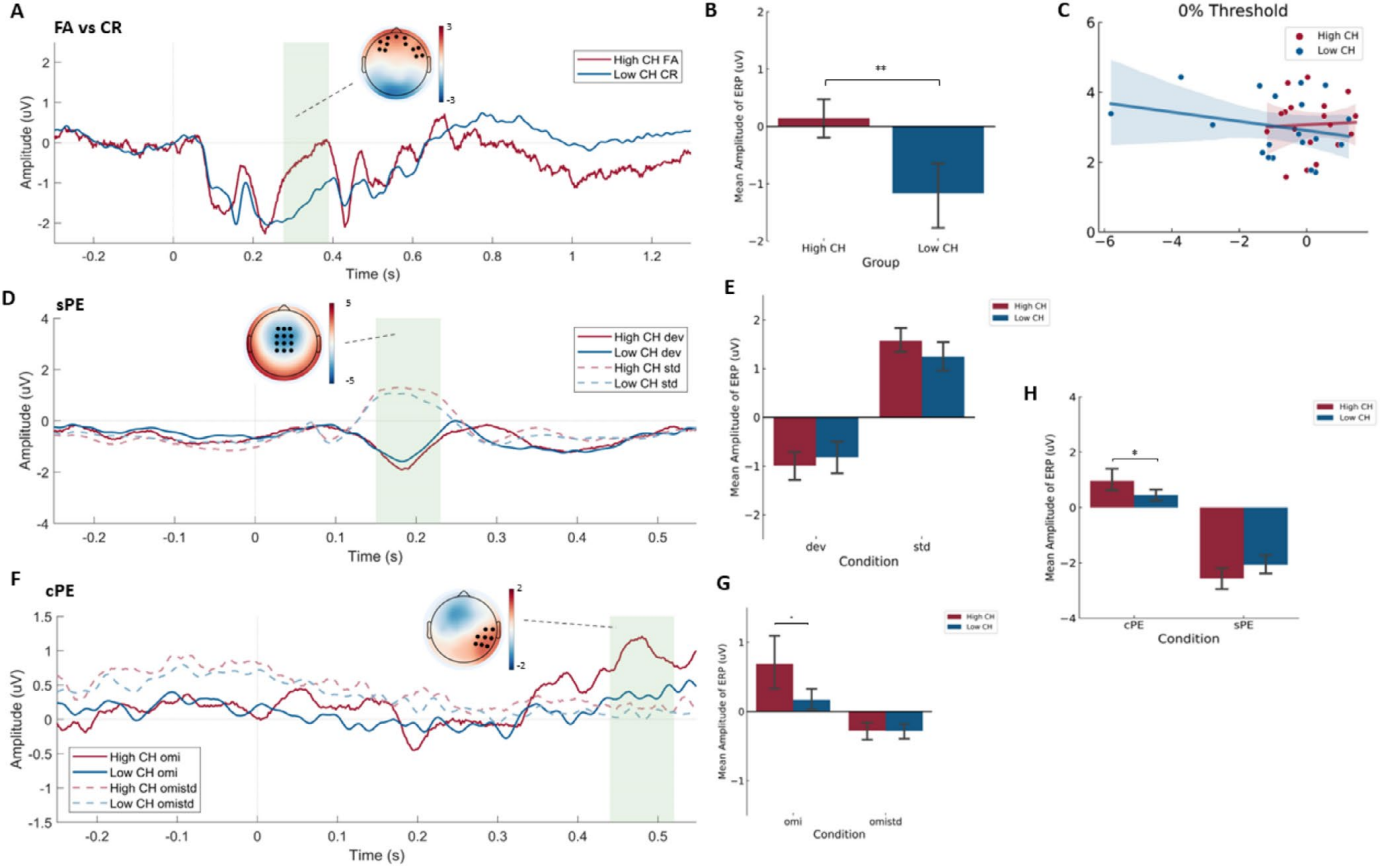
Between‐group Neural responses in the CH and auditory oddball tasks. The ERPs were grand averaged over the subjects in each group for each condition across the channels in black. The topographical plot represented the group or condition difference. The mean amplitude was averaged across the significant channels and timepoints (regions in green) between the two groups in the CH or two conditions in the oddball task. (A) shows the ERP and scalp plot of false alarm (FA) in high perceivers and correct rejection (CR) in the low perceivers, (B) shows the mean amplitude of FA and CR evoked ERP in the two groups, (C) shows the correlation between the confidence rating with mean amplitude of FA‐evoked and CR‐evoked ERP in the high and low perceivers respectively, (D) shows the ERP and scalp plot of the std. and dev in the high and low perceivers, (E) shows the comparison of the mean amplitude of std. and dev between the two groups, (F) shows the ERP and scalp plot of the omistd and omi in high and low perceivers, (G) shows the comparison of the mean amplitude of omistd and omi between the two groups, (H) shows the comparison of the context‐driven PE (cPE) and stimulus‐driven PE (sPE) amplitudes between the two groups. Data are presented as mean ± 95% CI. *p < 0.05; **p < 0.01.

When comparing the two groups, we observed no difference between the amplitude of the evoked response for the HT 75, 50, 25 between the two groups (cluster‐based *p* value > 0.05). However, we observed a significant increase in the amplitude of the evoked response to the FA in the high perceivers compared to CR in the low perceivers in a cluster of frontal electrodes consisting of (Fpz, Fp1, Fp2, AF3, AF4, AF7, F3, F5, F6, F8, FT8, FC6) between 276 and 390 ms for more than 80% of comparisons (cluster‐based *t*_max_ = 3441.9, *p*_min_ = 0.002) (Figure 7A,B).

Based on the theory that the confidence of the perceptual decision is inversely related to its sensory cortical responses, we hypothesized that the amplitude of the evoked response for the FA and CR would be negatively correlated with the confidence of this perceptual decision. In line with this hypothesis, we observed that the amplitude for the evoked response for the high perceivers was negatively correlated with that of the confidence (*R* = −0.466, *p* = 0.026). This was not observed for the low perceivers (*R* = 0.037, *p* = 0.440). Furthermore, there was a significant difference in the steepness of the slopes between the two groups (*t* = 2.068, *p* = 0.047) (Figure 7C). This provided further evidence that the high perceivers relied less on the external input and more on the internal model (i.e., make strong priors) and the low perceivers relied more on the external input and less on the internal model.

##### 3.1.3.2 The Role of PEs in Manipulating Internal Models

A greater reliance on the internal model would mean the generation of stronger predictions of the incoming input. Evidence for the high perceivers making strong priors was provided by the results of the auditory local–global paradigm. When the evoked responses to the standard (std) were compared to that of the deviant (dev) in the whole group, we observed a significant change in the mis‐match negativity timeframe (150–230 ms) in the frontal‐central electrodes consisting of (Fz, F1, F2, FCz, FC1, FC2, Cz, C1, C2, CPz, CP1, CP2) as seen in the classic oddball literature (Garrido et al. 2009) and the original study of the paradigm (Wacongne et al. 2011) (cluster‐based *t*_max_ = 1604.7, *p*_min_ < 0.001) (Figure 7D). When the amplitude at the significant channel‐timepoint cluster was averaged for the std. and dev and compared between the two groups, we observed no significant group × condition effect (*F*(1,35) = 3.326, *p* = 0.077, partial *η* ^2^ = 0.087) (Figure 7E).

We then compared the evoked responses to the expected (omistd) and unexpected (omi) omission sequences. We observed a significant temporal–parietal cluster consisting of electrodes (i.e., C6, T8, CP4, CP6, P4, P6, P8 and TP8) in the late positive potential timeframe (440–520 ms) similar to what was shown in the original paradigm (cluster‐based *t*_max_ = 3313.9, *p*_min_ < 0.001) (Figure 7F). When the amplitude at the significant channel‐timepoint cluster was averaged for the omi and omistd and compared between the two groups, we observed a significant group × condition interaction (*F*(1,35) = 4.970, *p* = 0.032, partial *η* ^2^ = 0.124). Posthoc comparisons revealed a significantly increased amplitude for the omi condition in the high perceivers compared to the low perceivers (*F*(1,35) = 5.694, *p* = 0.023, partial *η* ^2^ = 0.140), but no difference for the omistd between the two groups (*F*(1,35) = 0.003, *p* = 0.959, partial *η* ^2^ < 0.001) (Figure 7G). This suggests that a change in the probability/context of the same stimulus invoked a higher degree of surprise in the high perceivers, indicating increased sensitivity to the deviance of the prior in this group.

This was seen more clearly when the average sPE (std—dev) and cPE (omi—omistd) amplitudes were compared between the two groups. We observed a significant group × PE interaction (*F*(1,35) = 7.713, *p* = 0.009, partial *η* ^2^ = 0.181) with no difference in the amplitude of sPE between the two groups (*F*(1,35) = 3.326, *p* = 0.077, partial *η* ^2^ = 0.087) and increased amplitude of cPE in the high perceivers compared to the low perceivers (*F*(1,35) = 4.970, *p* = 0.032, partial *η* ^2^ = 0.124) (Figure 7H). These results showed that the high perceivers were as sensitive as the low perceivers to PEs driven by surprises in stimulus characteristics but were more sensitive to changes in the context of the same incoming stimulus. This provided evidence for the hypothesis that the high perceivers build strong priors for the same stimulus, which when deviated results in increased context‐related PE in neural responses.

The SHS is a measure of a subjective evaluation of how often people think they perceive false percepts in everyday life. We observed a significant positive correlation between SHS and FA rate (*R* = 0.416, *p* = 0.01). However, this relationship was not significant after controlling for the 75% detection threshold (*R* = 0.238, *p* = 0.163). This could act as preliminary evidence for future studies to comprehensively evaluate the role of constructs like self‐awareness and personality in the generation of false percepts.

## 4 Discussion

The current study provides an empirical estimation of the different components of the predictive coding system (subjective confidence, priors, PE, internal model) and how they interact with one another to form a perceptual inference. Here, we study how these different components interact with one another in three steps by investigating the role of (i) subjective confidence in building priors, (ii) priors in building an internal model, and (iii) PEs in manipulating the internal model during a perceptual inference.

### 4.1 Role of Subjective Confidence in Building Priors and Biasing Perception

Subjective confidence is hypothesized to originate from metacognitive processes that provide a subjective measure of one's beliefs (Sanders et al. 2016). Confidence is shown to be a reliable measure to determine if changes in decision reflect changes in perception (Gallagher et al. 2019). The relationship between subjective confidence and perceptual decision making in a noisy environment is not one‐to‐one (Balsdon et al. 2020; Zylberberg et al. 2016). Increasing the ambiguity of the environment lowers accuracy, decreases reaction times, and increases confidence in perceptual decisions in both monkeys and humans (Zylberberg et al. 2016).

In the current study, we observed that in the high perceivers, confidence in a perceptual decision may act as a reinforcement learning strategy to associate the target auditory signal with the visual cue when the signal was reliable. Such an effect has been observed in other tasks where confidence enhanced perceptual learning (Guggenmos et al. 2016) and was found to be a fundamental feature driving decision in an uncertain environment (Kepecs et al. 2008). As the uncertainty of the signal increased, we observed that the accuracy of the whole group decreased and they were more confident in reporting the absence of a signal relative to the presence of the signal.

However, on a comparative basis, the high perceivers were more likely to report the inaccurate presence of the stimulus during the stimulus‐absent trials, with relatively more confidence than the low perceivers. We see this pattern start to emerge when the stimulus is presented at a 50% threshold (i.e., equally probable and improbable at being perceived). The increased confidence in this evidence is in line with the literature that counterfactual confidence (i.e., confidence in equally probable perceptual outcomes) strengthens the formation of perceptual priors (Zylberberg et al. 2018), and this increase in strength of the prior has been shown to be instrumental in the generation of false percepts (Corlett et al. 2019). The perceptual decision towards the perception of the target, even in its absence, when cued by the visual signal is also in line with the research that shows that these decisions may be biased towards the prior (Feigin et al. 2021)–that is perceiving the two together. As evidence, we observed that in the high perceivers, the relative confidence was strongly related to making this decision when the signal was detectable (75% condition) and when the signal was absent, not when the signal was unreliable (50%, 25%). Furthermore, their preference to answer “yes” to the absence of the stimulus is negatively related to the 75% detection threshold, suggesting that they may be more reliant on the internal model itself than the sensory stimulus.

However, self‐reported confidence has been shown to be influenced by personality traits and response bias. An early study showed that traits of extroversion and extraversion were positively correlated with increased confidence (Kim et al. 2006). Furthermore, it has also been shown that high confidence in an initial perceptual decision may bias an observer to accumulate evidence inducing a selective gain towards that decision, resulting in a confirmation bias (Rollwage et al. 2020). Given these dependencies of confidence in addition to perceptual certainty, it is required by future studies to take this into account while studying perceptual predictive coding processes.

From the perspective of the low perceivers, we observed that they have higher perceptual thresholds than the high perceivers, suggesting they may have a system that is more reliant on the stimulus itself rather than the internal model. This is also seen in the relationship between the 75% threshold and response preference, wherein their relative preference to say “yes” to an absent stimulus is not dependent on the threshold. Furthermore, we also observed that they were more confident in reporting the accurate absence of the target signal, which suggests that their confidence in their belief and choice may allow them to explore the environment more than exploit the knowledge they already gained, as opposed to what the high perceivers may be doing (Boldt et al. 2019).

This exploration‐exploitation trade‐off was further evidenced with the results of the ERP. The increase in amplitude for the FA‐evoked ERP in the frontal region is in line with the literature that suggests the role of the prefrontal region and inferior frontal region in causal inference and perceptual decision making in a multisensory environment (Cao et al. 2019; Gao et al. 2023; Weilnhammer et al. 2021; Gherman and Philiastides 2018). This amplitude was inversely related to the confidence of making this decision in the high perceiver group, suggesting that high confidence may reduce sensory responses since the response may be dependent more on the internal model (exploitation) (Mulders et al. 2023). By contrast, the lack of a predictive relationship between confidence and amplitude of CR‐evoked ERP suggests the dependence on the external signal (exploration). Furthermore, the differences in the ERPs of hits and misses at the different threshold levels in the whole group were observed to be localized to the central parietal electrodes. This is also in line with the multisensory learning literature showing that integration of audiovisual signals is being localized to regions including the temporal–parietal junction and superior temporal gyrus in the central‐parietal regions (Scheliga et al. 2023).

### 4.2 Role of PEs in Shaping Internal Models

Building internal models is an efficient way to navigate changing sensory environments (Friston 2010; Barra et al. 2010; Lee 2015). It provides a predictive strategy to expect the incoming sensory input (Knill and Pouget 2004) thereby reducing the effort on the peripheral system to constantly scan the environment. However, building overly strong internal models is shown to have a maladaptive effect leading to chronic phantom perceptions (Hullfish et al. 2019; Drusko et al. 2023), hallucinations (Corlett et al. 2019) and proneness to psychosis in healthy individuals (Stuke et al. 2021). The changes in this predictive internal model (PEs) can be measured by probing the hierarchical predictive coding system of the brain using the local–global paradigm (Bekinschtein et al. 2009), as shown by previous studies (Mohan et al. 2022; Uhrig et al. 2014; Candia‐Rivera et al. 2023). PEs play a vital role in learning the environment (Den Ouden et al. 2009; Holland and Schiffino 2016; Behrens et al. 2007). In the local–global paradigm performed in the current study, responses to predictable stimuli (std, omistd) represent learning the environment and building the internal model, and the responses to deviations from this prior (dev, omi) represent responses to sensory uncertainty. Deviations driven by changes in stimulus (sPE) are seen as an increase in MMN, and deviations driven by changes in context (cPE) are seen as an increase in P300, replicating the original results (Wacongne et al. 2011).

From the results of the local–global paradigm in the current study, we observe that both high and low perceivers build an internal model that records a stimulus and context‐related deviation from it. However, what is interesting is that the deviation in the context (cPE) in the high perceivers was higher than that in the low perceivers, whereas there was no difference between the two groups for stimulus‐driven differences (sPE). Contextual information can be extracted from the configuration of sensory information to facilitate the recognition of a target stimulus in the current environment (Kveraga et al. 2007; Sanocki 1993). This is important to build the internal world for perceptual inference (Aminoff et al. 2013). Although there is a violation of an internal model in both sPE and cPE, the violation of the expected regularity of the same stimulus (cPE) represents a change in a higher‐order process that cannot be explained by changes in stimulus characteristics, thereby reflecting on a purely probabilistic model. The change in this probabilistic model in the high perceivers alludes to the evidence of a stronger probabilistic model in this group.

### 4.3 Future Applications

Although the current study explored a purely sensory environment, the results may be applied to study other cognitive and semantic contexts in information processing. With the advent of social media, we live in a world of sensory overload. Information is repeatedly brought to us through multisensory media, without a lot of filter of what is real and what is fake. It will be interesting to study the effect of subjective confidence in encoding multisensory information with a specific context to observe the changes in behavioral and electrophysiological signals of those who accurately perceive the news as real or fake. From our exploratory analysis, we found preliminary evidence to show that subjective knowledge of previous hallucinations was related to the objective measure of CH. However, when accounted for their individual perceptual thresholds, this relationship no longer existed. Therefore, there may be a complex interplay between signal detection and higher‐order cognitive processes that result in the decision making of perceptual inferences and bias the predictive coding system of the brain in one way or the other. These findings may also be extended to studies interested in identifying a subsection of the population who may be at a higher risk of developing chronic maladaptive perceptual inferences when either the sensory system and/or the top‐down inhibitory systems are damaged (Vanneste et al. 2019).

### 4.4 Limitations

The biggest limitation of the current study is still that the results are based on a theoretical model that lacks empirical proof. This study is one of the first attempts to bring these different components of the predictive coding system together so as to provide future studies an empirical base to test their data on. The results of the current study need to be replicated by future studies and using computational models to ensure the empirical validity of its results.

## 5 Conclusion

In summary, the current study demonstrated how different components of the predictive coding system, such as subjective confidence, priors, PEs, and internal model, interact with each other to produce a percept using illusion as a perceptual model. It explores the role of confidence in building strong priors, which, when conflicted, result in false perceptions of the world, and the role of these strong priors and PEs in shaping and updating the internal model. We showed that people who are more likely to perceive false perceptions depended more on their internal model rather than the external input. Furthermore, they were more sensitive to changes in context compared to those less likely to perceive false perceptions, thereby showing the influence of strong priors on perceptual inference.

## Author Contributions

**Feifan Chen:** experimental design, data collection, data analysis, and writing manuscript. **Anusha Yasoda‐Mohan:** experimental design, data analysis, and writing manuscript. **Colum Ó Sé:** data collection and data analysis. **Sven Vanneste:** ideation, supervision, and writing manuscript.

## Conflicts of Interest

The authors declare no conflicts of interest.

## Data Availability

The data that support the findings of this study are available on request from the corresponding author. The data are not publicly available due to privacy or ethical restrictions.
